# Identifying Early Life Habitat of Golden Mahseer Fish *Tor putitora* (Hamilton, 1822) in South Asia: Implications for Conservation

**DOI:** 10.1002/ece3.72117

**Published:** 2025-09-17

**Authors:** Anu Rai, Bibhuti Ranjan Jha, Kundan Lal Shrestha, Elio Guarionex Lagunes‐Díaz

**Affiliations:** ^1^ Department of Environmental Science and Engineering School of Science, Kathmandu University Dhulikhel Nepal; ^2^ Sustainability and Environmental Studies Endeavor (SENSE) Kathmandu Nepal; ^3^ Instituto de Ecología Veracruz Mexico

**Keywords:** distribution modeling, early life habitat, evidence‐driven conservation, golden mahseer, Himalayan mahseer, nursing, spawning, threats, *Tor putitora*

## Abstract

The endangered golden mahseer ( *Tor putitora* ) (Hamilton, 1822) is a famed gamefish that has a restricted area of occupancy in the Himalayas. Owing to overexploitation, hydrologic alteration along rampant riverbed mining in its spawning grounds, both its number and size have significantly declined. A lack of study on the habitat status of the species is hindering the identification of local habitat threats. Planning evidence‐driven conservation demands the identification of critical habitats and local habitat of golden mahseer. Hence, the overall aim of the study was to ascertain the habitat suitability of golden mahseer in its early life stage. For this, Species Distribution Modeling was conducted using spawning and nursing occurrence records. These habitats were mostly restricted to the northern region of its range. Almost 90% of the priority area identified as spawning and nursing habitat by this study is currently unprotected. With the identification of such habitats, conservation intervention can be further planned.

## Graphical abstract

The occurrence records of golden mahseer into early life stages: Spawning and nursing and has successfully delineated the current distribution of such habitats across its entire range. Almost 90% of the priority area identified as spawning and nursing habitat by this study is currently unprotected. Our findings stress the importance of community‐based conservation interventions and the necessity to address multiple stressors, including channel modifications and fishing practices.

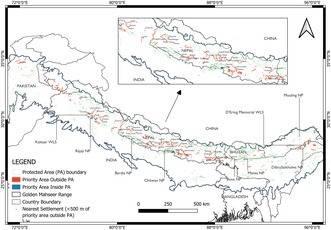

## 1 Introduction

A famed gamefish, the golden mahseer, also known as the Himalayan mahseer ( *Tor putitora* ) (Hamilton, 1822) has a restricted area of occupancy in the Himalayas. Its massive size, strength, culinary quality, elusive nature, attractive golden color, and resilience have made it fascinating for anglers and consumers. Specimens as large as 2.75 m and 54 kg have been recorded (Everard and Kataria 2011; Nautiyal et al. 2008). However, overexploitation and a host of other stressors, such as the loss of connectivity, riverbed mining, and pollution, have significantly declined the population size of the species. It is estimated to have declined by > 50% in the past 21 years, and the current business as usual scenario may well plunge the population further (Jha et al. 2018). Hence, it is categorized as Endangered in the IUCN Red List (Jha et al. 2018).

The golden mahseer has different ecological requirements in its life phases. It is a migratory species using small hill streams for breeding or spawning and large rivers for feeding. Overall, the species showcases a phased migration in the Himalaya with three phases of migration occurring: first phase as learning behavior, second phase for spawning, and third phase as descent to larger streams (Nautiyal 1994). The first phase is undertaken by adults during March–April by ascending to higher tributaries from larger rivers. However, the small tributaries are not entered as the water level is not adequate. The second phase of migration is undertaken by brooders during the onset of monsoon as the water becomes turbid. The monsoonal rain swells up these streams and makes it optimal to sustain brooders of golden mahseer. The fish spawn in these tributaries. It requires these habitats to increase the chances of hatching its eggs and to ensure less predation (Bhatt and Pandit 2016). As this phase is completed, it returns to the larger rivers where the species can grow massively owing to large food availability and large volume of water. However, meeting these ecological requirements has become a problem.

Indiscriminate and over‐exploitative fishing practices such as the use of dynamites, poisons, or the diversion of water for fish collection in nonprotected areas are major threats to the golden mahseer (Nautiyal 2014). Being a prized game fish, the species is subject to extensive overfishing practices. The asymptotic length (*L*_*∞*_) of the species has shown a rapid decline, with *L*_*∞*_ being 272 cm in the early 1980s to *L*_*∞*_ declining to 216 cm in the mid‐1990s (Nautiyal et al. 2008) and presently, the largest fish recorded by anglers in the decade have not exceeded 150 cm (Jha et al. 2018).

Likewise, hydrological alterations such as dams obstruct the longitudinal connectivity of the golden mahseer by obstructing continuous flows and creating isolated habitats (Yadav et al. 2020). Dams can also fragment the golden mahseer populations by creating barriers to their movement and disrupting their breeding and migration patterns (Naik et al. 2024). Currently, 652 hydropower plants have been constructed, 162 are under construction, while 720 hydropower plants have been planned in the Himalayan rivers (Hennig et al. 2023), posing a serious challenge for the conservation of the species.

Similarly, riverbed mining activities for extracting sand and gravel cause major destruction in its spawning sites (Bhatt and Pandit 2016; Jha et al. 2018). These substrates are required for laying its eggs. The introduction of exotic species has also led to a decline in the population of golden mahseer. For instance, in the Govind Sagar reservoir, major reductions in the mahseer population were noted after the introduction of an exotic species—silver carp (Molur and Walker 1998). Besides, the inflow of pesticides has been shown to retard or cease food uptake in the golden mahseer (Kunwar et al. 2021). Furthermore, an increase in temperature brought on by climate change is likely considered to impact its growth and health status (Akhtar et al. 2014).

Conserving the species requires conserving its different habitat requirements. The use of a bio‐surveillance tool has been limited but has revealed great insights; for example, in the Manas watershed in Bhutan, the species has been able to attain upstream movements of 30 km in just 24 h periods, with fishes showcasing homing behavior to distinct tributaries (cited in Pinder et al. 2019). This goes to highlight much that is unknown in its other distribution range. However, the lack of extensive studies in many rivers has hindered evidence‐based conservation actions. As the species is increasingly threatened, it is imperative to map the habitats of the species for conserving the occupied habitat as well as for identifying potential habitats for interventions such as translocation (Jha et al. 2018). But again, resource constraints make it difficult to implement interventions throughout.

Urgent conservation efforts will have to be initiated to save the species from extirpation in several locations (Jha et al. 2018). Planning evidence‐driven conservation demands the identification of critical habitats and local habitat conditions of the golden mahseer (Bhatt and Pandit 2016). Hence, the overall aim of the study was to ascertain the habitat suitability of golden mahseer during its early life stages (spawning and nursing) phase in South Asia. The specific objectives were:

- To map the habitat suitability of the golden mahseer during its spawning and nursing stages.
- To prioritize areas for its conservation and research
- To assess the threats the species faces in selected sites.

## 2 Methods and Materials

### 2.1 Study Area

The presence of golden mahseer has been noted in South and Southeast Asian countries—Nepal, India, Bangladesh, Afghanistan, Pakistan, Bhutan, and Myanmar. Its longitudinal distribution extends from Hindukush–Kabul–Kohistan in the Northwest Himalaya to Sadiya (Brahmaputra) in the Northeast Himalaya. It is distributed naturally throughout the rivers and reservoirs of Indus, Ganges–Yamuna, and Brahmaputra—rivers of South Himalayan drainage (Jha et al. 2018). Naturally, the species inhabit high energy river systems with rapids and pools, but it can also persist in lakes where ephemeral stream input can provide breeding grounds (Bhatt and Pandit 2016; Jha et al. 2018). Its distribution mapping till date has been done in different regions and countries (Table 1). This study was conducted in the entire extant and possibly extant range of golden mahseer as compiled by Bournemouth University which is also followed by the IUCN Red List (Jha et al. 2018). The region forms part of the Hindu Kush Himalaya (HKH) which is an origin of 10 major river basins and is considered a critically important geo‐ecological region (Sharma et al. 2019).

**TABLE 1:** Distribution mapping of golden mahseer.

Area	Type of study	Data used	References
Bhutan	Distribution mapping	Superimposing the occurrence records into elevation and temperature data	Wangmo et al. (2022)
North of India to south of Nepal	Mapping migratory routes	River network and occurrence records	ADB (2019)
Karnali basin, Nepal	Identifying the spawning and nursery area of fishes	Species occurrence records and presence time. Plus, expert consultation.	USAID (2020)
Karnali river, Nepal	Demarcation of conservation zone	Expert consultation	NRCT (2019)
From Jammu and Kashmir in the west till Bhutan	Distribution modeling	Occurrence plus environmental records	Mahato et al. (2022)
Kosi and Kolhu streams of the Ramganga basin	Generalized Linear Modeling (GLM) to analyze environmental characteristics.	Occurrence plus environmental records	Johnson et al. (2020); WII (2021); Dhawan et al. (2023)

**FIGURE 1:**
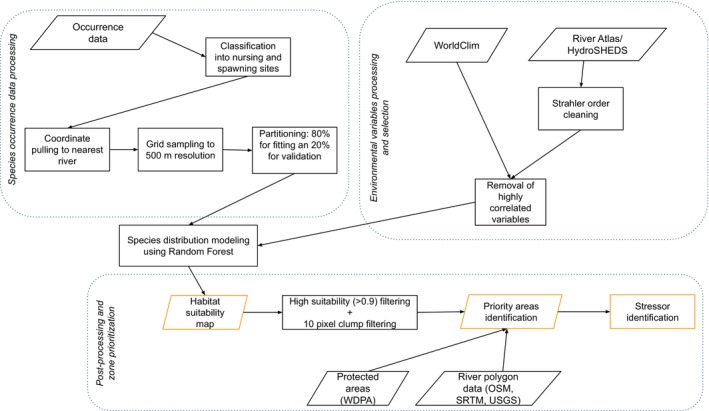
Schematic diagram of the data used and methods involved.

The research follows a multistep methodology from occurrence data collection and modeling using different environmental variables and final identification of conservation priority sites based on habitat suitability (Figure 1).

### 2.2 Occurrence Data

**FIGURE 2:**
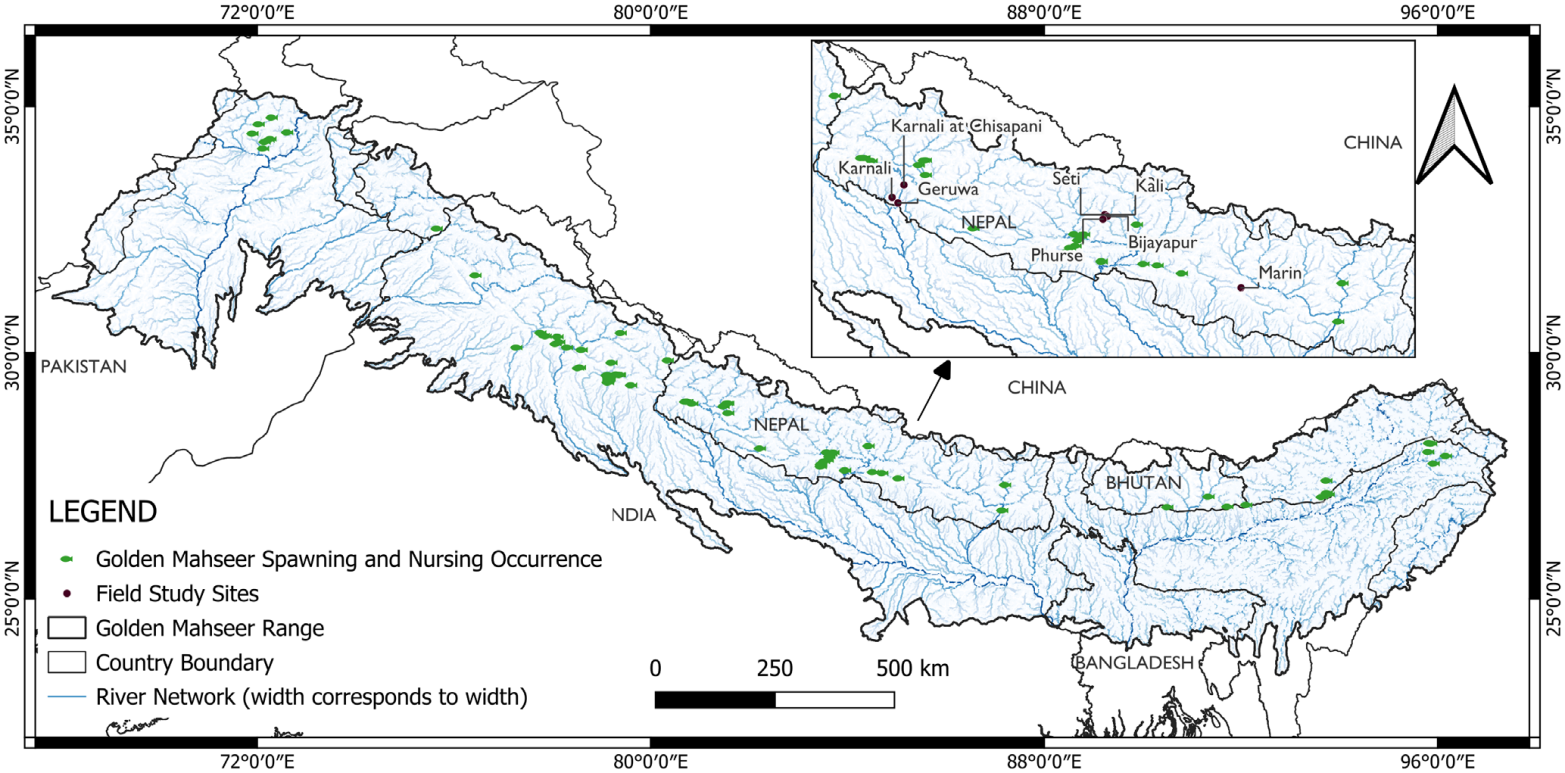
Early life occurrence records of golden mahseer in both its extant and possibly extant ranges.

The occurrence records of golden mahseer were collected from various online repositories, reports, research papers, personal records of researchers, etc. (Figure 2). Specific search terms using a combination of keys: golden mahseer, *Tor putitora* , occurrence, GPS, latitude, longitude, coordinate, location, <range countries name> or their derivatives were used in the search for these records in Google Scholar and Google. From these searches, records from 16 sources were obtained (Jha 2006; Shafi 2010; NGFGR 2013; Khare et al. 2014; CWC 2015; Philipp and Claussen 2015; Sati et al. 2015; Johnson et al. 2020; Yousaf 2021; DAI Global LLC 2021; Dwivedi 2021; WII 2021; Khan et al. 2021; Attaullah et al. 2022; Mahato et al. 2022) including GBIF.org (2025) which itself is a compilation of 24 contributing sources for the species.

Within coordinates available in the Global Biodiversity Information Facility (GBIF) if the coordinate uncertainty was mentioned to be greater than 1000 m and/or coordinates with an event date before 1990 were discarded. The coordinates occurring in reservoirs and dams were also discarded. In total, 317 occurrence records of the species were collected. The records were classified as either spawning and nursing sites or not to keep only sites of early life stages for priority sites of conservation. The classification of the sites was made based on a review of literature, time of occurrence, stream order, etc. For a point to be classified as spawning or nursing sites, it had to meet the following requirements (Table 2):

**TABLE 2:** Criteria for classification of coordinates for spawning and nursing sites of golden mahseer.

Criteria	Details and examples
Site identified as spawning or nursing grounds in the paper itself or occurrence in rivers already identified as spawning grounds	WII (2021) identified nursing sites of the species using telemetry techniques. Likewise, the Song river has been identified as an important spawning ground for the species (Bhatt and Pandit 2016) and the occurrence of coordinates at Song river led to its classification as a spawning site
River segments of Strahler order up to 4	Golden mahseer requires lower order streams for spawning. However, since the exact order of the stream used for spawning has not been identified, the reference to previous studies which conducted ecological studies which identified habitat suitability of golden mahseer young ones was used and using GIS the order of those streams were extracted either as point value or 500 m coordinate pull if the value was null (Johnson et al. 2020; WII 2021; Dhawan et al. 2023)

Timing of occurrence was not found to be an appropriate way of classifying the sites as golden mahseer are known to spawn multiple times as long as their ecological requirements are met (Bhatt and Pandit 2016). Out of these records, coordinates which did not have environmental values to model potential distribution of golden mahseer were moved to the nearest raster cell within a river within 500 m using terra and RANN package. The geographic coordinates obtained were again grid sampled to match the raster resolution. This resulted in 84 occurrence points used in modeling spawning and nursing sites. Pseudoabsence selection is advised when no true absences are available or when they are not numerous enough (Thuiller et al. 2025). Since the true absence data is not available, pseudoabsence was generated as equal weightage to the presence points.

### 2.3 Environmental Variables

Sixty‐four environmental variables were obtained from a range of databases which include WorldClim, HydroSHEDS, and RiverATLAS/HydroSHEDS (Lehner 2013; Fick and Hijmans 2017; Linke et al. 2019). A 15 arc‐second spatial resolution of variables was used through rasterized using the v.to.rast module. WorldClim datasets were resampled using the r.resamp.bspline module, and bilinear method in GRASS GIS 8.3.2. Lapse rate correction was also applied for resampling.

Before rasterization, stream segments of Strahler order 1 touching segments greater than Strahler order 2 were omitted as these segments caused error in the continuous rasterization of higher order streams. This omission was done using disjoint feature selection in the “Select by location” feature in QGIS. Ecologically relevant variables having least correlation (−0.75 > correlation < 0.75) were selected for distribution modeling using Pearson correlation matrix which resulted in the use of five variables for modeling which are flow length in upstream direction, human footprint, stream gradient, mean temperature of warmest quarter, and precipitation of wettest quarter (Table 3).

**TABLE 3:** Predictor variables used to model the distribution of golden mahseer.

Variables	Description	Range in the study
Flow length in upstream direction (hyd)	Flow length to the furthest source of the river	0 to 3211.570 km
Human footprint (hft)	The human footprint in the reach catchment (year 2009). This sums together eight factors, including built environments, population density, electric infrastructure, crop lands, grazing lands, roads, railroads, and navigable waterways, to indicate the relative human effect on the environment globally	0 to 482
Stream gradient (sgr)	Ratio between the elevation drop to the length of the river reach	0 to 9108 decimeter/km
Mean temperature of warmest quarter (BIO10)	Mean temperature that prevails in the warmest quarter	−8.93°C–34.83°C
Precipitation of wettest quarter (BIO16)	Total precipitation that prevails in the wettest quarter	85–6051.188 mm

### 2.4 Distribution Modeling and Statistical Validation

A single modeling approach has been used in this study using “biomod2” package, version 4.2‐4 (Thuiller et al. 2025) in RStudio Version 2025.05.0. Random Forest (RF) which is a classification and machine learning algorithm, was used for modeling. It classifies new objects from an input vector that are positioned underneath each tree. These trees then contribute to prediction making. In modeling aquatic species such as fish, algorithms such as RF have been used owing to different properties of the algorithm (Buisson et al. 2008; Olaya‐Marín et al. 2013). The choice of algorithm used and the configuration settings used in SDM algorithms greatly impact the results of species distribution (Hallgren et al. 2019). In this study, model performance was evaluated using the committee averaging score (Emca).

Model selection was done using True Skill Statistic (TSS) evaluation metric with a threshold of 0.7. Each model was evaluated by TSS and area under curve (AUC) metrics. Before conducting SDM, data partitioning (80% for fitting and 20% for evaluating) was conducted. Variable importance was assessed statistically using the function variables_importance from “biomod2” package, which compares the prediction of the reference value versus the prediction with its values shuffled by subtracting 1 minus the correlation of both predictions.

### 2.5 Identification of Priority Areas for Conservation and Research

The distribution model obtained was used to identify areas with a high probability of species occurrence. These areas were then overlaid on protected area networks in the range countries. The boundaries of protected areas were obtained through the World Database on Protected Areas. Additionally, India's protected areas were supplemented by a database obtained from WII (2015). This helped in calculating the extent of areas of high probability of species occurrence currently under protection and the extent which are outside.

The identified clumps (patches) of connected cells showing high habitat suitability were then filtered into river segments, which has also been done in the case of other aquatic species (Rai et al. 2023). Clump detections were performed for cells with very high probability of species occurrence (values with > 0.9 probability) considering eight directions for cell adjacency and cell frequency greater than 10, which means > 10 cells with > 0.9 probability of occurrence need to be connected from any of the eight directions to be considered a clump.

OpenStreetMap (OSM) data were imported in R with OSM key natural features of river, water, and waterway. Likewise, Shuttle Radar Topography Mission (SRTM) data sources were obtained from the United States Geological Survey (USGS). These were merged, dissolved, and subdivided with a maximum of 1000 vertices to create a comparable area to generate river polygons for identifying priority areas in rivers. The nearby settlements have also been obtained from OSM data using key = place and value = village, hamlet, isolated_dwelling, suburb, quarter, neighborhood, city_block, locality, city, or town_square.

### 2.6 Habitat Assessment and Identification of Local Stressor

Assessment of water quality at selected sites was conducted using a calibrated handheld multiparameter probe, Extech DO700: Portable Dissolved Oxygen Meter by Extech. Five water quality parameters were noted: pH, water temperature, conductivity, total dissolved solids (TDS), and dissolved oxygen (DO). These measurements were compared with a range of habitat preference of the species obtained through literature review.

Field observations were used in identifying stressors on the species in Karnali, Geruwa, Marin, Seti, and its three tributaries Kali, Bijayapur, and Phurse at different periods between September and November 2022. The stressors on habitats were observed at each site and classified into five broad categories: solid wastes, effluents, activities and facilities, hydromorphological and ecological degradation, and sanitation on the basis of criteria given by Shrestha et al. (2009) (Table 4). The stressors were identified in situ by visual observation and with consultation with residents and local people. Stressor categorization is helpful in the identification of broad stressor categories, and it provides valuable information for prioritizing conservation action plans at local to watershed scales.

**TABLE 4:** Stressors and their potential impacts.

Stressor category	Stressor component	Potential impacts
Solid wastes	Waste dumping and cremation activities	Leads to water quality degradation which could affect reproductive health and stress (Abass et al. 2024)
Effluents	Sewage, agricultural effluent, industrial effluent, and landfill leachate	Leads to riverine pollution such as metal contamination which could result in physiological and behavioral changes, reproductive disruption among others (Hashim et al. 2024)
Activities and facilities	Squatter settlements, picnic spots close to river vehicles crossing along rivers or using rivers as roads and littering by picnic goers	Could lead to dumping of untreated waste and littering which could degrade water quality and impact their health
Hydro morphological and ecological degradation	Channel, embankment and weir, bank cutting, reservoir, dam and impoundment, irrigation, fishing & boating, stone quarrying & crushing, and sand quarrying	Hydrological impediments impact migration and riverbed mining activities destroy breeding grounds (Naik et al. 2024)
Sanitation	Bathing & washing and practice of open defecation	Could introduce surfactant and increase organic/nutrient load

## 3 Results

### 3.1 Distribution Modeling, Their Performance, and Factors Influencing the Distribution Patterns

**FIGURE 3:**
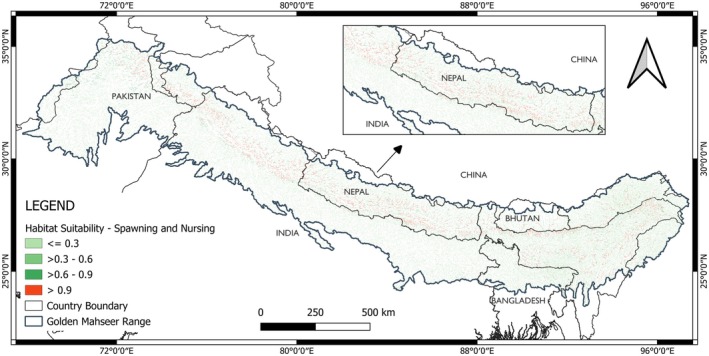
Model output of golden mahseer habitat suitability for early life stages.

The study shows the potentially suitable spawning and nursing habitat is concentrated in the northern region of its range (Figure 3). The majority of the highly suitable habitat occurs in India, followed by Nepal. The spawning and nursing habitats include the tributaries of the Karnali River in Nepal, Tongsa River in Bhutan, Nayar River in India, and many other watersheds in its range countries.

Overall, the model (EMca) has shown a good AUC and TSS score. For modeling of spawning and nursing phase, the AUC score of Random Forest ranged from 0.998 to 1.000, AUC with a median score of 0.999. The model has also shown a good TSS score ranging from 0.955 to 1.000 and a median score of 0.985.

**FIGURE 4:**
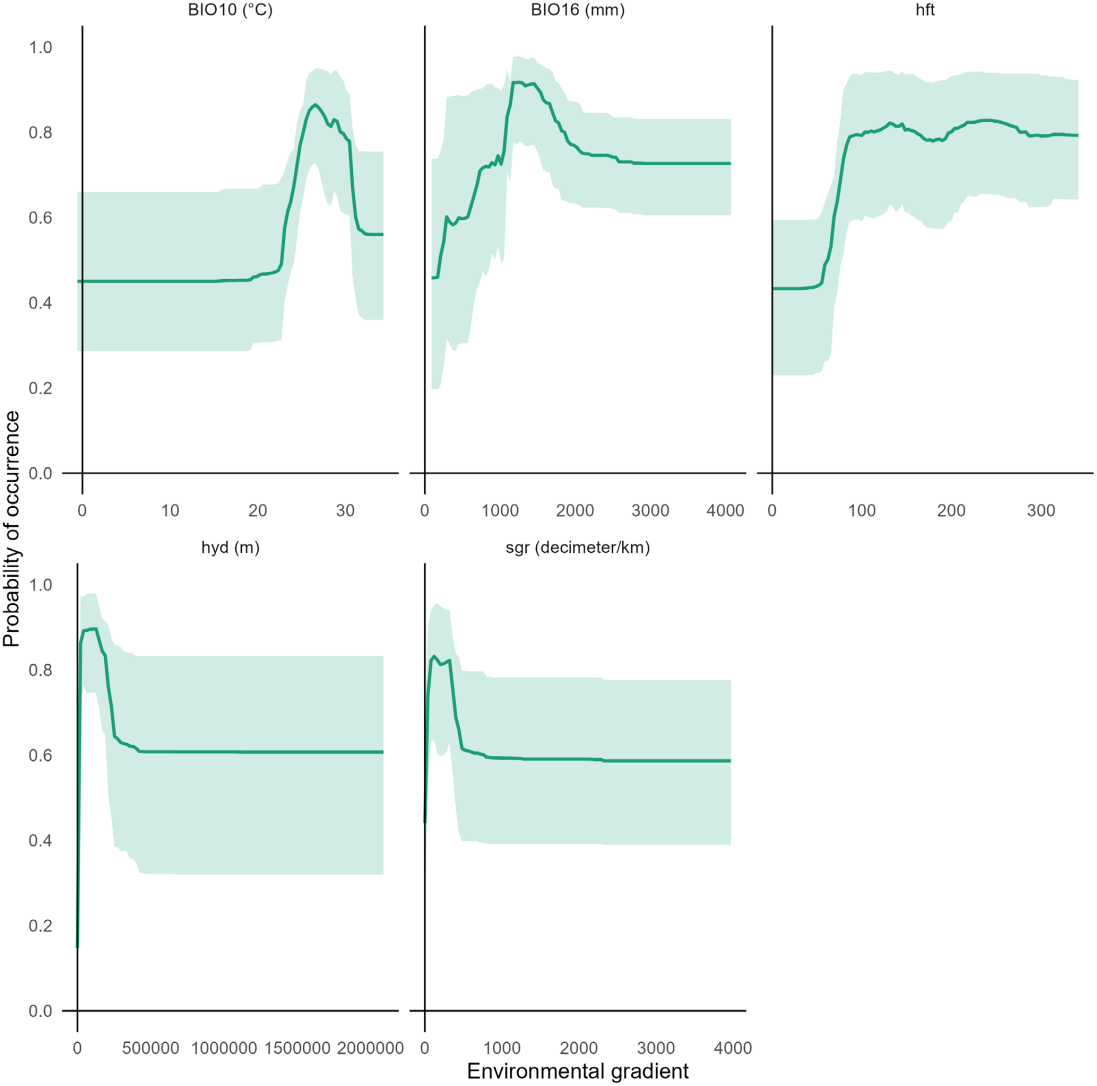
Response curves of species distribution model.

Flow length in upstream direction, stream gradient, and mean temperature of warmest quarter were found to be the most important variables for modeling. High probability (> 0.9) of golden mahseer occurrence was observed between flow length in upstream direction of around 21 km to 210 km with an interquartile range of around 63 km to 147 km, stream gradient of around 40 to 320 decimeters per km with an interquartile range of around 120 to 270 decimeters per km, and mean temperature of warmest quarter from 25.5°C to 29.7°C with an interquartile range of 26.9°C to 28.7°C (Figure 4).

### 3.2 Priority Areas for Conservation and Research of Early Life Stages of Golden Mahseer

**FIGURE 5:**
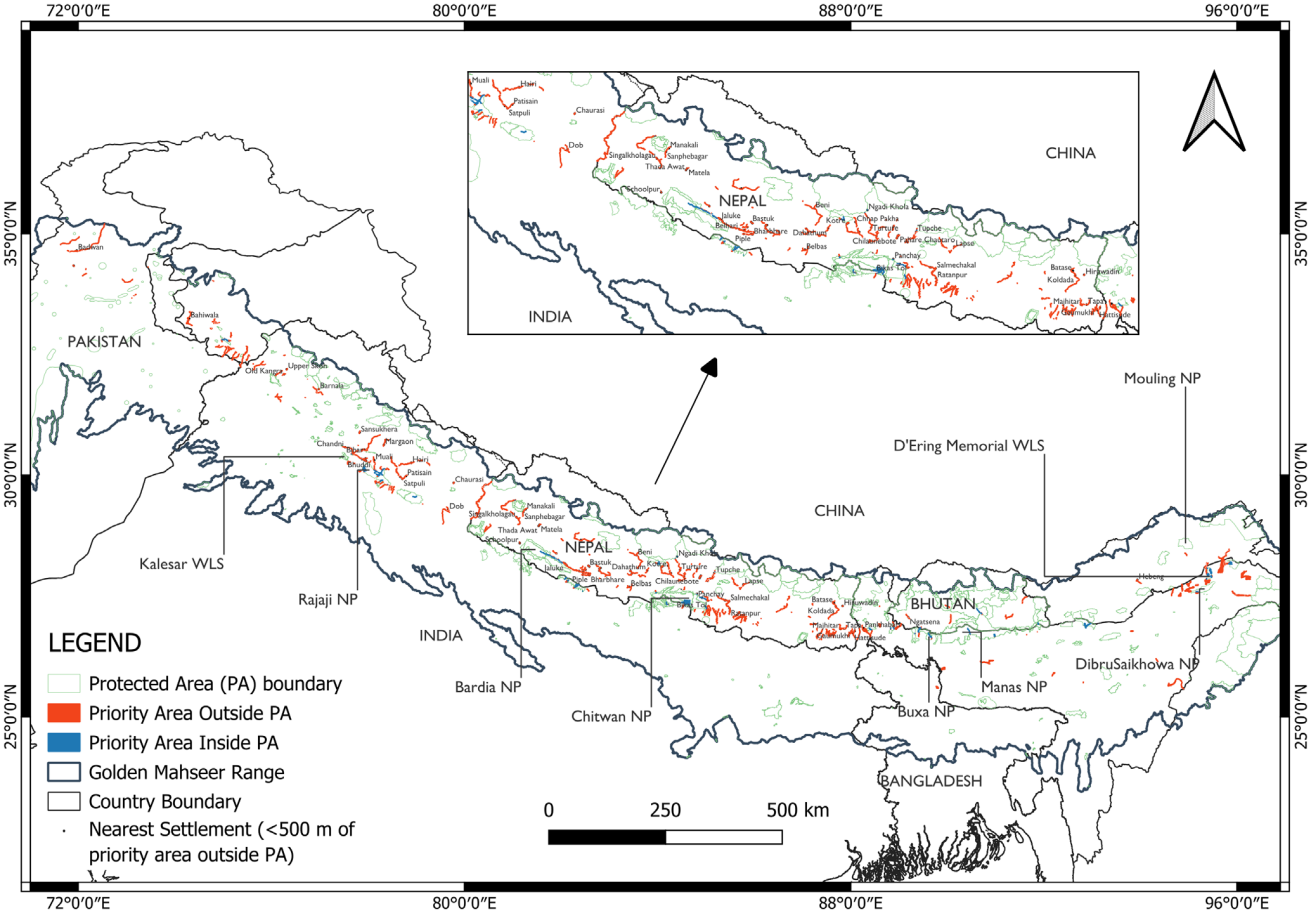
Priority areas for conservation and research of golden mahseer. PA, protected area; NP, national park; WLS, wildlife sanctuary.

A significant portion of the priority areas (~90%) identified as spawning and nursing habitat by this study is currently unprotected (Figure 5 and Table 5). The priority areas for conservation and research have been identified for Nepal, India, Bhutan, Pakistan, and Bangladesh. Overall, India has the highest priority areas of early life stages of golden mahseer for both protected and unprotected areas. Priority areas inside Pakistan and Bangladesh inside protected areas have been shown to be zero, but this does not mean they do not have golden mahseer protected habitat. It can mean two things: either the size of the connected habitat suitability areas (identified by the model) was not large enough to be considered clumps or the river segments were not represented as polygons or protected areas boundaries. For instance, river segments in the Poonch River lying in Pakistan had been identified as clumps; however, because of the lack of river segment polygons and protected area boundaries in those areas, the priority area was not selected.

**TABLE 5:** Priority areas for conservation and research of early life phases of golden mahseer.

Areas (sq. km)	Entire range	Nepal	India	Bhutan	Pakistan	Bangladesh
Total	1319.12	215.05	1013.63	8.37	71.89	10.17
Inside Protected Areas	144.55	17.29	126.5	0.77	0	0
Outside Protected Areas	1174.56	197.76	887.14	7.60	71.89	10.17

### 3.3 Habitat Assessment and Identification of Local Stressor

The hydro morphological and ecological degradation was the most observed common stressor that occurred in the form of construction of structures for irrigation and use of toxin for fishing. In Marin khola, the locals described that golden mahseer was found until a few years back, but after rampant use of poison for fishing, it is no longer being found in the area. Fishing using toxins was apparently quite common after the decline in water depth, as reported by the residents in Geruwa River. It has only been a few years since such activities were stopped. Locals at Majhara, lying in Geruwa River, informed community educational programs against the use of toxins on fishing, leading to a decline in its use. Likewise, Karnali river supports Rani Jamara Kulariya Irrigation Project, which has seen the development of intake and embankment along its stretch. The other stressors observed were effluents, mainly in the form of agricultural effluent; personal hygiene and sanitation in the form of bathing and washing; and activities and facilities such as vehicle washing and vehicle crossing along the river.

The physicochemical parameters were found mostly within range. The pH ranged from 7.8 to 8.7, exhibiting a slightly alkaline nature of water. Golden mahseer also prefers alkaline pH (Bhatt and Pandit 2016) so the range is well within the bounds. The stream temperature ranged from 14.9°C to 28.8°C, with the readings taken during the day. Golden mahseer has a wide range tolerance to temperature, with preference within 13°C to 30°C (Joshi et al. 2018). The conductivity and TDS have ranged between 177.2 and 418 μS and 118 and 282 mg/L, respectively. These readings are well within the bounds considered normal limits for golden mahseer (Johnson et al. 2020). Likewise, DO of some sites is lower, occurring within 5.62 to 9.7 mg/L than the range defined between 6.4 and 11 mg/L in breeding grounds (Bhatt et al. 2004; Joshi 1994).

## 4 Discussion

### 4.1 Predicted Golden Mahseer Distribution and Suitable Habitats, and Their Status

The secondary source data validates the predicted golden mahseer distribution. The model classification and later field validation of the Marin River as breeding grounds (although currently not found due to rampant use of poison) provide primary validation. Likewise, the Tadi and Khahare khola (tributaries of Trishuli River) in Nepal (Shrestha 2002), the Korang River in Pakistan (Zafar et al. 2002), the Nayar River in India (Bhatt et al. 2004), and the Dangme‐Chu and Mangde‐Chu Rivers in Bhutan (WWF Bhutan 2019) are considered spawning grounds of the golden mahseer. Despite the lack of occurrence points in these sites, the model output has considered these sites as high suitability areas, providing validation through the secondary source data.

The predicted distribution of golden mahseer has varying probabilities of occurrence throughout its range. The highly suitable areas have discrete availability. The golden mahseer completes its life phase broadly into two distinct habitats: the upstream smaller streams and the downstream larger rivers. The specific requirements for spawning and nursing came to be as a way to increase the survival of its eggs and young (fry and fingerlings) from predators and to prevent the sinking of its eggs in the muddy lowland rivers. However, this has also made the species at higher threat as dams and barrages impede their migration and riverbed mining activities destroy their breeding grounds. This highlights an urgent need for targeted conservation of the species.

However, the decision pertaining to conservation of the species, especially in terms of e‐flows, mostly pertains to its adult life stage with an assumption that this will be sufficient for the young life stage as well. But, as pointed out by Nale and Pakhale (2024) the habitat requirements of the young are not always captured well. Integrating these into conservation planning is essential to ensure the species' long‐term survival.

### 4.2 Hotspots for Golden Mahseer Conservation and Research

Certain river segments are more important than others when it comes to prioritization, which makes the identification of these areas important from the point of conservation and research. This rationale is applied as an effective conservation tool seen in both marine and, in certain cases, freshwater fishery management. Prohibiting fishing in specific locations through the regulation of marine protected areas (MPAs) has been assessed as an effective tool for conservation and fishery management (Harmelin‐Vivien et al. 2008). Whereas the conservation of small disconnected segments in rivers has also led to enhanced species biomass, density, and richness in rivers (Koning et al. 2020).

The priority areas identified in this research are to be taken as the initial step for designing a conservation intervention for the species (Figure 5). Though the networks seem disconnected, a spillover from the reserve has been assessed to enhance local fisheries; for instance, in Thailand's Salween basin, an average of 2247% higher biomass, 124% higher fish density, and 27% more fish species were found through the assessment of grassroots reserves in comparison with nonreserve areas (Koning et al. 2020). The capacity of these reserves, however, depended on several factors such as the reserve area, distance to village, years protected, and distance to mouth. Despite the small size of the networks, these have enhanced higher fish biomass, density, and richness. Hence, there is a need to initiate community‐based conservation intervention at high‐probability areas of its occurrence.

Yet dedicated regions for freshwater ecosystems are rare except in some cases such as Ramsar sites, and mostly the efforts piggyback on terrestrial region protection. However, even small grassroots reserves have proven to be a great conservation strategy. As specific river segments are of more priority than others, the identification of these areas is important from the point of conservation and research. This research can also be combined with distribution modeling of other fish species, and in combination can facilitate the identification of loose networks of community‐managed reserves with input from other variables such as centrality metrics.

### 4.3 Methodological Constraints and the Way Forward

Though an extensive effort was undertaken for the collection of occurrence records, records from many locations remained inaccessible. However, the records of the extent of the species' occurrence were obtained and represent the largest pool of the occurrence records of the species compiled till date. The classification of the species occurrence was done into early life stages. However, in the truest ecological sense, the classification should be done separately for breeding and nursing (separately for juveniles and fingerlings). However, spawning and nursing grounds have a high degree of overlap (DAI Global LLC 2020) as the early life form of golden mahseer is not capable of negotiating strong currents during floods (Nale and Pakhale 2024).

Likewise, there are many environmental variables that remain unavailable currently. Spawning habitats are characterized by riverbeds with pebbles, gravels, and large boulders. Likewise, high DO is required for both of its spawning grounds. Besides, the use of biotic factors in modeling, such as prey of fish, such as macroinvertebrates, could enable better prediction of the model. But these environmental variables are not available for modeling. Hence, the model developed in this research is only the first step towards a comprehensive study on identifying the best priority areas for its conservation. The modeling outcomes should not be considered an exact representation of the underlying datasets and should therefore be interpreted with appropriate context.

Besides, much field‐based research is required to validate the overall predictions made by the model; to some extent, field validations have been done, and further continuity of this work is required. Besides, the polygons of the river networks and the protected areas are not available throughout the range of the species, which has hampered identifying priority areas for its conservation.

## 5 Conclusion and Recommendation

Our study has classified the occurrence records of golden mahseer into early life stages: Spawning and nursing and has successfully delineated the current distribution of such habitats across its entire range, with a concentration in the northern region. The identified factors influencing high probability of distribution patterns, such as flow length in the upstream direction of around 21 km to 210 km, with an interquartile range of around 63 km to 147 km, have contributed valuable insights for understanding the species' habitat preferences.

The identification of priority areas for conservation and research is a crucial outcome of our study. The majority of these areas currently lack protection, and as water development projects are rapidly undergoing in their range countries, it is imperative to prioritize conservation actions. Despite challenges in certain areas, such as in Pakistan, where the lack of represented river polygons and/or protected area boundaries affected the assessment, the study underscores the urgency of safeguarding these critical habitats. Field validations and insights from local communities have further highlighted the impact of human activities, such as the use of toxins in fishing, on golden mahseer populations.

Riverscapes have in general been neglected in designing protected areas. However, declaring protected areas for the species will hardly be a solution for the conservation of the species as riverscapes encompass large areas and are transboundary in nature. Hence, community‐based conservation programs must be given priority for the conservation of the species with a necessity to address multiple stressors, including channel modifications and fishing practices. In summary, our research provides a foundation for identifying priority areas, understanding habitat preferences, and formulating conservation strategies for golden mahseer. Overall, our research emphasizes the ongoing need for collaborative efforts, extensive field studies, and adaptive management to ensure the effective conservation of this iconic species in South Asia.

## Author Contributions

**Anu Rai:** conceptualization (lead), data curation (lead), investigation (lead), methodology (equal), project administration (lead), software (lead), validation (equal), visualization (lead), writing – original draft (lead). **Bibhuti Ranjan Jha:** investigation (equal), supervision (lead), writing – review and editing (equal). **Kundan Lal Shrestha:** investigation (equal), methodology (equal), writing – review and editing (equal). **Elio Guarionex Lagunes‐Díaz:** methodology (equal), software (equal), visualization (equal).

## Conflicts of Interest

The authors declare no conflicts of interest.

## Supporting information


**Data S1:** Supporting Information S1.

**Data S2:** Supporting Information S2.

**Data S3:** Supporting Information S3.

## Data Availability

The data used for this research were derived from the following resources available in the public domain:
WorldClim (https://worldclim.org/) HydroSHEDS (https://hydrosheds.org/) RiverATLAS (https://www.hydrosheds.org/page/hydroatlas) The outputs from species distribution modeling and priority area identification are available in the Supporting Information.
